# The Relationship between In-Training Examinations and Simulation Assessments in an Emergency Medicine Residency Program

**DOI:** 10.51894/001c.4941

**Published:** 2016-10-24

**Authors:** Sheri L. Clarke, Ali Eydgahi

**Affiliations:** 1 Director of Medical Education McLaren Greater Lansing Hospital, Lansing, MI; 2 School of Engineering Technology Eastern Michigan University, Ypsilanti, MI

**Keywords:** assessment, simulation, in-training examination, emergency medicine

## Abstract

**CONTEXT:**

Historically, the assessment of medical resident knowledge and skill has occurred through annual in-training examinations and faculty observation during real patient encounters. To improve patient care and the learning environment, medical educators have begun creating simulation experiences for medical residents to perform procedures without using real patients. However, simulation curricula and accompanying assessment techniques have not been standardized. Using a longitudinal record review, in-training examination scores were compared to annualized simulation assessment scores to see if there is any relationship between the assessment types.

**METHODS:**

This project was a retrospective eight-year study from a single residency program. The scores were collected from 102 resident academic records from 2007 to 2015 for the annual American Board of Emergency Medicine in-training examination and the resident’s annual simulation assessment. Complete data sets were analyzed to determine if a relationship exists between the assessment methods. Then the averages were compared for only the first three years for all students and for all four years for osteopathic residents as they have an additional fourth year of training.

**RESULTS:**

This study showed a lack of relationship between the two assessment types when reviewing three years of data. When the fourth year data is considered, there was a significant relationship between the assessment types.

**CONCLUSIONS:**

The performance scores for both types of assessment provide independent information on the resident progress in training. Therefore, they should both be reviewed and considered to appropriately measure the resident’s performance. The significance of the fourth year of training for osteopathic residents requires further study.

## INTRODUCTION

Assessment of medical residents’ knowledge and skill level is often done through subjective evaluations by faculty and by using objective standardized written examinations. In traditional residency training, clinical training occurs with faculty observation of real patient encounters. While this practice may be an effective educational technique for training the next generation of physicians, it puts patients at risk.^1^

To improve patient care and the learning environment, medical educators need to expand beyond traditional training and assessment methods.^2^ Simulation allows residents to practice and improve their technical skills while working on their cognitive development in a safe and non-threatening environment.^3^ Residency programs across the country are already using hybrid training models in which both simulation and traditional training are employed.

Annual in-training examinations are the historical way to assess trainee medical knowledge, whereas simulation is the newest assessment technique that encompasses both medical knowledge and clinical skills. It is necessary to determine whether these assessment tools equally identify competency or provide different independent scores that should both be considered in the assessment of competency for each trainee.

**Table 1.** Relationships Identified in the Literature for Different Types of Assessments

	COMLEX-1	COMLEX-2	IN-TRAINING	BOARD CERTIFICATION	PERFORMANCE IN RESIDENCY	BOARD REVIEW/CONFERENCES	SIMULATION
USMLE-1	7+		15* 16* 18+ 19+ 20+ 5- 22- 9# 11# 21#	15* 8+ 10+	12+ 17-		
USMLE-2		7+	16* 18* 20* 5+ 19+ 22- 11# 21#		17-		
USMLE-3			16* 18+				
IN-TRAINING			16*	15* 8+ 10+ 13+	12+	6- 14-	
CLINICAL PRODUCTIVITY			13-	13-			
FACULTY ASSESSMENT			4-		4+		
INDIVIDUAL EDUC PLAN				23+			
SIMULATION							

There is a large amount of literature that address standardized assessments in multiple medical specialties, but no literature was found that addresses simulation scores and their relationship to standardized written examinations.^4–23^ A summary of the most relevant literature is provided in Table 1. In Table 1, numbers represent corresponding papers listed in the references section, * denotes strong positive relationship, + shows relationship, - represents no relationship, and # indicates that poor performance on one assessment is predictive of poor performance on the other. As indicated in Table 1, the impact of the use of simulation technology for assessment has not been sufficiently explored.

This study provided an analysis of the in-training examination scores and the annualized simulation scores to determine if there is any relationship between these assessment methods. The focus of the study was only on emergency medicine residents in a single program.

## METHODS

This project was a retrospective study using a quantitative research method. The focus of this project was on a dually accredited residency program, which consists of osteopathic and allopathic residents. The residency program has an average of 34 residents with 10 new residents added to the program each year. This study included a sample of 102 individual residents from 2007 to 2015. The study was designed as the census of a single residency program in Lansing, Michigan to ensure that all residents in the selected sample had received the same training, used the same trainers and simulation center for all of their training, and had similar patient experiences. To improve the sample size, the study was longitudinal, with data collected for each resident in the program over an eight-year period. The institutional review board for the sponsoring hospital approved the study in affiliation with the university where this was part of a larger project for a doctoral student dissertation. Test scores were collected from the residents’ academic records for the annual American Board of Emergency Medicine (ABEM) in-training examination and the residency program’s annual simulation assessment. The score identified for each individual was the percentage of questions answered correctly with 100 being the maximum possible score.^24^ The in-training examination is valid, as it is a national standardized assessment that was designed to assess a resident’s knowledge of learning objectives set by the ABEM.^25^ The scoring for the examination is reliable, as it is a standardized examination administered by the ABEM.

The simulation scores were collected using evaluation tools that were developed by the residency program faculty eight years ago. Multiple simulation scenarios and multiple assessments of similar scenarios have been combined to create an annual simulation score for each year in the program. Each of these scores were listed as a percentage of correct answers with a maximum of 100 points.

The evaluation scores were assigned by one of five faculty members. The faculty has demonstrated high intra-rater and inter-rater reliability over the past eight years (unpublished data). The faculty member that moderates the simulation session scores the resident. All scores and video of the simulation are reviewed and verified by a second faculty member prior to being entered into the database. The simulation assessments are presumed to be valid because they were developed by board certified attending physicians in Emergency Medicine to assess the ABEM learning objectives. The scores are presumed to be reliable because they represent eight years of data collection, where each resident has been evaluated with the same tools through multiple observations over multiple years.

In order to review and analyze the assessment methods for a relationship, the data points were separated into subsets of data. These data were identified by year of training using post graduate year (PGY). Because this was a dually accredited program, osteopathic residents who were dually enrolled in the American Osteopathic Association (AOA) and Accreditation Council for Graduate Medical Education (ACGME) had four years of data, while all other residents enrolled only in the ACGME program had three years of data. Therefore, differences in the training years were also considered by examining only the first three years of training, as well as the full data set for the osteopathic residents. Data was identified as In-Training Examination (ITE-1, ITE-2, ITE-3, ITE-4), Simulation (Sim-1, Sim-2, Sim-3, Sim-4), Average of all scores (AvgITE and AvgSim), and the first three year average of all scores (AvgITEx3 and AvgSimx3).

Canonical correlation was determined to be the appropriate method for analysis as it uses correlation coefficients and weighted sums for all potential interactions to determine significance of relationships between all data subsets in a single analysis.^26^ For this type of study, using canonical correlation has several benefits over using multiple regression. It allows the researcher to review relationships with fewer calculations, but it also decreases the risk of Type I error by decreasing the number of regression equations required for analysis.^27^ Variables can be either metric or nonmetric and must have at least 10 measurements per subset in order to have an acceptable sample size.^27^ It is important to note that the correlation method does not support claims of cause and effect. It just determines whether or not the variables have a relationship. In order to infer causality, further experimental studies would need to be completed.^28^

In this study, the scores of all participants were collected to create data sets for each individual trainee. Complete data sets were analyzed in StatGraphics Software, using canonical correlation to determine if a relationship exists between the assessment methods.^29^ The averages were then compared using two sample comparisons in the statistical software.

## RESULTS

The simulation scores (Sim1, Sim2, Sim3, and Sim4) and in-training examination scores (ITE1, ITE2, ITE3, and ITE4) were reviewed using canonical correlation analysis. There were 14 identified complete cases within this data. Four reviews of the variables were completed with a P-value of 0.5 and higher as shown in Table 2. This was interpreted as having no statistically significant relationship between the data sets for DO residents.

**Table 2.** Canonical Correlation Analysis for Simulation and In-Training Examination Data Sets (All four years of data)

n=14		Canonical	Wilks			
Number	Eigenvalue	Correlation	Lambda	Chi-Square	D.F.	P-Value
1	0.530255	0.728186	0.245451	11.9396	16	0.7481
2	0.299442	0.547213	0.52252	5.51727	9	0.7871
3	0.214192	0.462809	0.745863	2.49231	4	0.6460
4	0.0508327	0.225461	0.949167	0.443447	1	0.5055

A second analysis using simulation scores (Sim1, Sim2, and Sim3) and in-training examination scores (ITE1, ITE2, and ITE3) was performed to compare only the first three years of scores to make sure non-osteopathic residents are included in the analysis. There were 50 identified complete cases within this data. Three reviews of the variables revealed a P-value of 0.29 and higher as demonstrated in Table 3. This can be interpreted as having no statistically significant relationship between the data sets.

**Table 3.** Canonical Correlation Analysis for Simulation and In-Training Examination Data Sets (Three years only)

n=50		Canonical	Wilks			
Number	Eigenvalue	Correlation	Lambda	Chi-Square	D.F.	P-Value
1	0.159308	0.399134	0.790233	10.712	9	0.2960
2	0.0585046	0.241877	0.939979	2.81634	4	0.5890
3	0.00161042	0.04013	0.99839	0.0733332	1	0.7865

The average Simulation score (AvgSim) and the average in-training examination score (AvgITE) were analyzed using two sample comparison methods to determine if they were significantly different. This is a comparison of all four years of data points. A summary of the data comparison is presented in Table 4. The report showed a Standard Skewness for AvgSim of -2.94, which indicates non-normal distribution and that comparisons based on standard deviation may not be valid.

**Table 4.** Summary Statistics for Average Simulation and In-Training Examination Scores

	All scores collected		Complete data sets only	
	Avg Sim	Avg ITE	Avg Sim x3	Avg ITE x3
Count (n)	94	102	51	64
Average	68.88	71.61	69.9	71.4
Standard deviation	9.82	5.75	5.18	4.98
Coeff. of variation	14.3 %	8.0%	7.40%	6.97%
Minimum	34.0	55.0	59.3	61.3
Maximum	92.0	86.0	89.3	84.7
Range	58.0	31.0	30.0	23.3
Stnd. skewness	-2.939	0.195	2.277	1.312
Stnd. kurtosis	5.562	0.137	4.679	-0.025

This indication of non-normal distribution led to a further analysis using Mann-Whitney U-test and Kolmogorov-Smirnov test as shown in Table 5. The Mann-Whitney U-test provides a way to compare the medians of the data sets on ordinal data.^30^ In this test a P-value of 0.02 indicated a statistically significant difference between the medians at a 95% confidence level. Then the samples were run through a Kolmogorov-Smirnov test to compare the distributions of the two samples.^31^ A P-value of 0.036 indicated a statistically significant difference between the two distributions at a 95% confidence level. These findings mean that the samples are not from similar groups, confirming that there is no relationship between the two groups, but not confirming or denying a relationship between assessment types.

**Table 5.** Median and Distribution Analysis for Average Simulation and In-Training Examination Scores from Table 4

	Median Analysis		Distribution Analysis	
	Mann-Whitney U-test		Kolmogorov-Smirnov test	
	P-value	Significantly Different	P-value	Significantly Different
Avg Sim / Avg ITE	0.0212178	Yes	0.0364329	Yes
(All scores collected) (n=51)				
Avg Sim x3 / Avg ITE x3	0.142311	No	0.0998673	No
(Complete data sets for first three years only) (n=64)				

P-value of less than 0.05 means that data sets are significantly different at 95% confidence level.

Next, the three year average Simulation score (AvgSimx3) and average in-training examination score (AvgITEx3) were reviewed to be consistent with the three year curriculum of the allopathic and international medical graduate residents. The three year data is only inclusive of complete data sets for the first three years of simulation and in-training examinations. A summary of the data comparison is shown in Table 4. The report showed a similar Standard Skewness for AvgSimx3 of 2.28, which indicates non-normal distribution and can invalidate comparisons based on standard deviation. This led to a comparison of medians using the Mann-Whitney U-test. In this test, a P-value of 0.142 indicated there is no statistically significant difference between the medians at a 95% confidence level. Then, the samples were run through a Kolmogorov-Smirnov test to compare the distributions of the two samples. A P-value of 0.09 indicated no statistically significant difference between the two distributions at a 95% confidence level. It should be noted that the three year average had a very different comparison result from the four year average as demonstrated in Table 5.

## DISCUSSION

For this single residency program, the data suggest there is no relationship between the performance of residents on the ABEM in-training examination and the program’s proprietary annual simulation assessment. However, when considering the first three year average scores, the samples are not significantly different, indicating a relationship between the scores. It appears that the first three years of training have similar assessment scores and the two assessment types are similar in nature. When the fourth year of training is added for the osteopathic residents, the scores are significantly different and no longer are related. The osteopathic residents’ scores are significantly different from those of the other residents, due to an additional year of training.

There has been a long discussion in the emergency medicine field as to whether the training should be three or four years.^32^ This additional year appears to make a significant difference in the relationship between the two assessment types. This could be important information for educators that are making decisions on whether emergency medicine training should be three or four years long.

## CONCLUSION

This study showed a lack of relationship between the two assessment types of in-training examination and the annual simulation assessment when reviewing three years of data. The fourth year data for the osteopathic residents raised further questions. When it was considered in the analysis, it showed that there were significant differences between the osteopathic residents and all other residents. When only the first three years of training was reviewed, there appeared to be no real difference between the different medical school types.

This raises a question as to why the fourth year data would make such a difference in the analysis. The authors believe that this can be explained by the fact that an additional year of data at the highest level of training is increasing the average scores for the osteopathic residents. If the resident was not providing higher scores in the fourth year, there would not be such a difference in the three and four year averages. Therefore, the performance scores for both types of assessment should be independently reviewed and considered to appropriately measure the resident’s performance. When the fourth year osteopathic data is considered, there is suddenly a significant relationship between the assessment types.

This research was limited to a single residency program over eight years of data collection. It was also limited to a specific simulation process that a single program has developed and implemented. Further research opportunities would include reviewing the same data from another program or multiple programs that have either similar or different simulation assessments.

In order to do further investigation on this topic, it may be beneficial to review individual resident performance in these assessments. Historically, those that do well on written examinations are thought to be more successful residents. Many times, board eligibility examination scores are used to filter applications when applying for residency. However, there are many people that have difficulty with written examinations, but excel in their field. Further research could follow individual residents to determine if those that demonstrate more knowledge on written examinations are the best at applying their knowledge in simulation.

The study has provided new information on the need to consider simulation assessment as an independent metric when reviewing resident performance. Many educators assume that those that do well in medical knowledge also do well in the application of that knowledge. This study has indicated that a direct relationship between the two assessment types does not exist in emergency medicine for this single residency program’s curriculum.

Another implication of this study, and any further research developed from it, is that it may assist in determining the best length of training for emergency medicine residency programs. Currently, the AOA requires a four year training program with an internship year and three years of emergency medicine training.^33^ The ACGME allows for either a three or four year length of program.^34^ With the unification of the AOA and ACGME accreditation systems, emergency medicine programs throughout the country may make significant changes in the training program length. For this particular dually accredited program, the plan is to eliminate the additional year of training for osteopathic residents as it unifies the accreditation of the programs. Does that additional year of training provide invaluable education, or are those physicians comparable to those with three years of training that spend the fourth year as an attending physician? That is the next question to answer. In order to answer that question, it would require a controlled study to analyze the simulation and in-training examination scores of the fourth year residents against the scores of the three year trained first year attending physicians. This would require a randomized sampling of residents and recent graduates across the country. It would require a significant investment in a standardized simulation assessment for all participants and a partnership with the American Board of Emergency Medicine in order to communicate with the attending physicians and collect the data necessary for comparison.

### Conflict of Interest

The authors declare no conflict of interest.
